# Cytological diagnosis of hyaline-vascular type of Castleman disease

**DOI:** 10.4322/acr.2024.519

**Published:** 2024-09-27

**Authors:** Deepa Rani, Anupam Varshney, Kanika Rastogi

**Affiliations:** 1 Sarojini Naidu Medical College, Department of Pathology, Agra, Uttar Pradesh, India; 2 Muzaffarnagar Medical College, Department of Pathology, Uttar Pradesh, India; 3 Atal Bihari Vajpayee Institute of Medical Sciences & Dr Ram Manohar Lohia Hospital, Department of Pathology, New Delhi, India

**Keywords:** Hyaline-vascular, Castleman Disease, fine needle aspiration cytology

## Abstract

Castleman disease (CD) is a rare, benign lymphoproliferative disorder, mostly involving the mediastinal lymph nodes, but can occur wherever lymphoid tissue is found. With only a few published case reports, there needs to be more literature on its cytological findings. We report the case of a 63-year-old female presenting with left upper cervical swelling. Fine needle aspiration cytology smears showed variably sized lymphoid follicles with diminished germinal centers, prominence of follicular dendritic cells, and capillaries traversing some of the follicles. The possibility of a hyaline-vascular type of Castleman disease was suggested. Histopathology confirmed the cytological diagnosis. The index case is being presented to discuss the cytological features of the CD along with its histological and immunohistochemical correlation.

## INTRODUCTION

Castleman disease (CD) is a benign lymphoproliferative disorder that may present as a nodal or extra-nodal mass. The exact etiology of this disease has largely remained unknown. However, infection by Human herpesvirus-8 and abnormal immunologic reactions resulting in elevated IL-6 levels have been hypothesized to be involved in the pathogenesis.^1-3^ Microscopically, two distinct histological patterns have been described- the hyaline-vascular type or angiofollicular type and plasma cell type.^4^ Though histopathological features of the disease are well described, the cytological diagnosis often remains challenging owing to morphological overlap with reactive and neoplastic lesions and a general lack of recognition regarding its cytological characteristics. Hence, we are presenting the cytomorphological features of a case of the hyaline-vascular type of CD, its histological and immunohistochemical correlation, and a brief review of the literature.

## CASE REPORT

**Figure 1:**
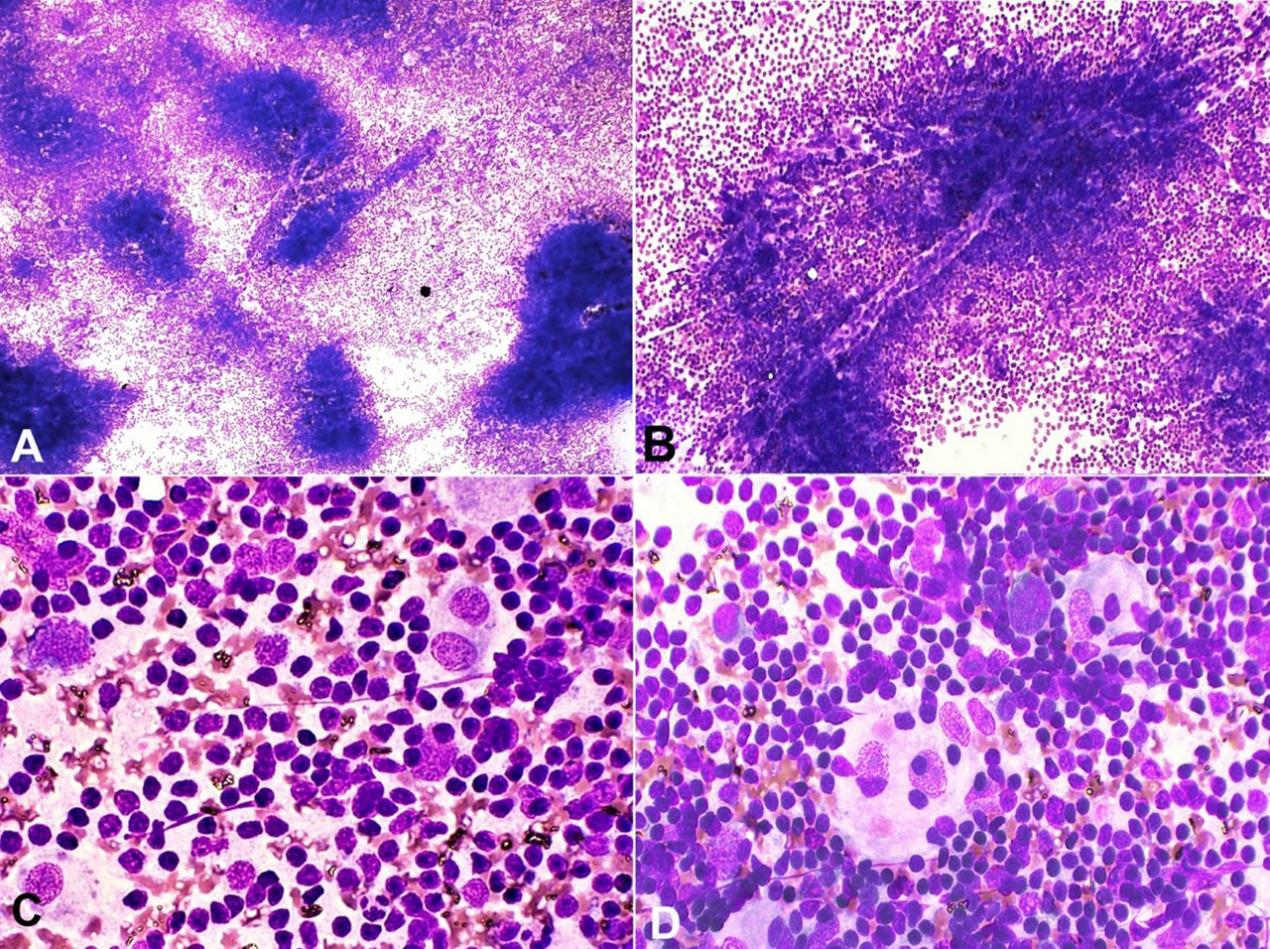
Cytological findings: FNAC smears. **A –** Show small, variably sized lymphoid aggregates (likely representing follicles) and a dispersed population of mature lymphoid cells (MGG, x100); **B** – A lymphoid aggregate comprising small lymphocytes (resembling diminished/atrophic germinal center) and traversing capillary fragment (MGG, x400); **C** – Follicular dendritic cells are seen in lymphoid follicles along with dispersed lymphocytes. The former are mononucleated and binucleated with finely granular chromatin, small nucleoli, and abundant pale cytoplasm (MGG, X400); **D** – Follicular dendritic cells showing emperipolesis (MGG, x400).

A 63-year-old female presented to the surgery outpatient department (OPD) of a tertiary care hospital with a slowly growing swelling in the left upper cervical region for 6 months. There was no history of upper respiratory tract infection, pain, fever, or weight loss. On physical examination, the mass was 4×3 cm in size, firm, mobile, non-tender, and not attached to the skin. The routine lab investigations were unremarkable. Ultrasonography demonstrated a hypoechoic oval mass measuring 4.3 × 3.3 × 2.1 centimeters in size, with increased vascularity, located in the anterior aspect of the upper neck on the left side. Fine-needle aspiration cytology (FNAC) was performed, and smears were stained with May-Grunwald Giemsa stain for evaluation. Cytology smears were cellular, comprising variable-sized nodular aggregates of small lymphocytes, which were traversed by capillaries and showed the presence of many follicular dendritic cells (FDCs), features resembling atrophic germinal center (Figures 1A and 1B). The FDCs were large in size and had abundant pale cytoplasm, single to multiple nuclei, finely granular chromatin, and small nucleoli (Figure 1C). Some FDCs contained mature lymphocytes in their cytoplasm (emperipolesis phenomenon) (Figure 1D). Based on these cytomorphological features, the diagnosis of a hyaline-vascular type of CD was suggested, and histopathological correlation was advised.

**Figure 2:**
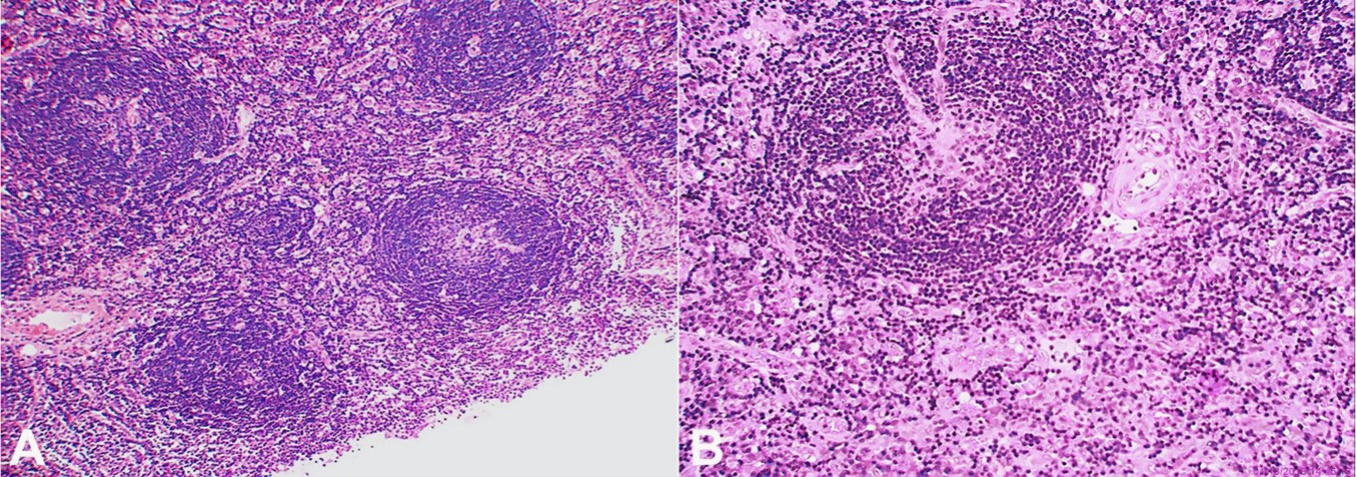
Histological findings: **A** – Lymph node showing an increased number of small-sized follicles with involuted germinal centers (H&E, x40); **B** – The germinal center shows hyaline deposits and a radially penetrating capillary. FDCs are increased and seen in the germinal center, within the concentric rims of lymphocytes, and in the interfollicular area. Many post-capillary venules surrounded by fibro collagenous tissue are seen in the interfollicular area (H&E, x100).

The excised mass was well-circumscribed and measured 4 × 3 × 2 cm. Microscopically, lymph node architecture was altered by increased lymphoid follicles (Figure 2A). The follicles were small, and germinal centers were involuted, poorly cellular, and showed hyaline deposits with radially penetrating capillaries. Small germinal centers were surrounded by multiple concentric layers of lymphocytes (Figure 2B). An increased number of FDCs was seen in the germinal centers, interfollicular region, and entrapped between the concentric rings of lymphocytes (Figure 2B). The interfollicular region also showed the presence of numerous post-capillary venules lined by hyperplastic endothelial cells and surrounded by fibro collagenous tissue (Figure 2B).

**Figure 3:**
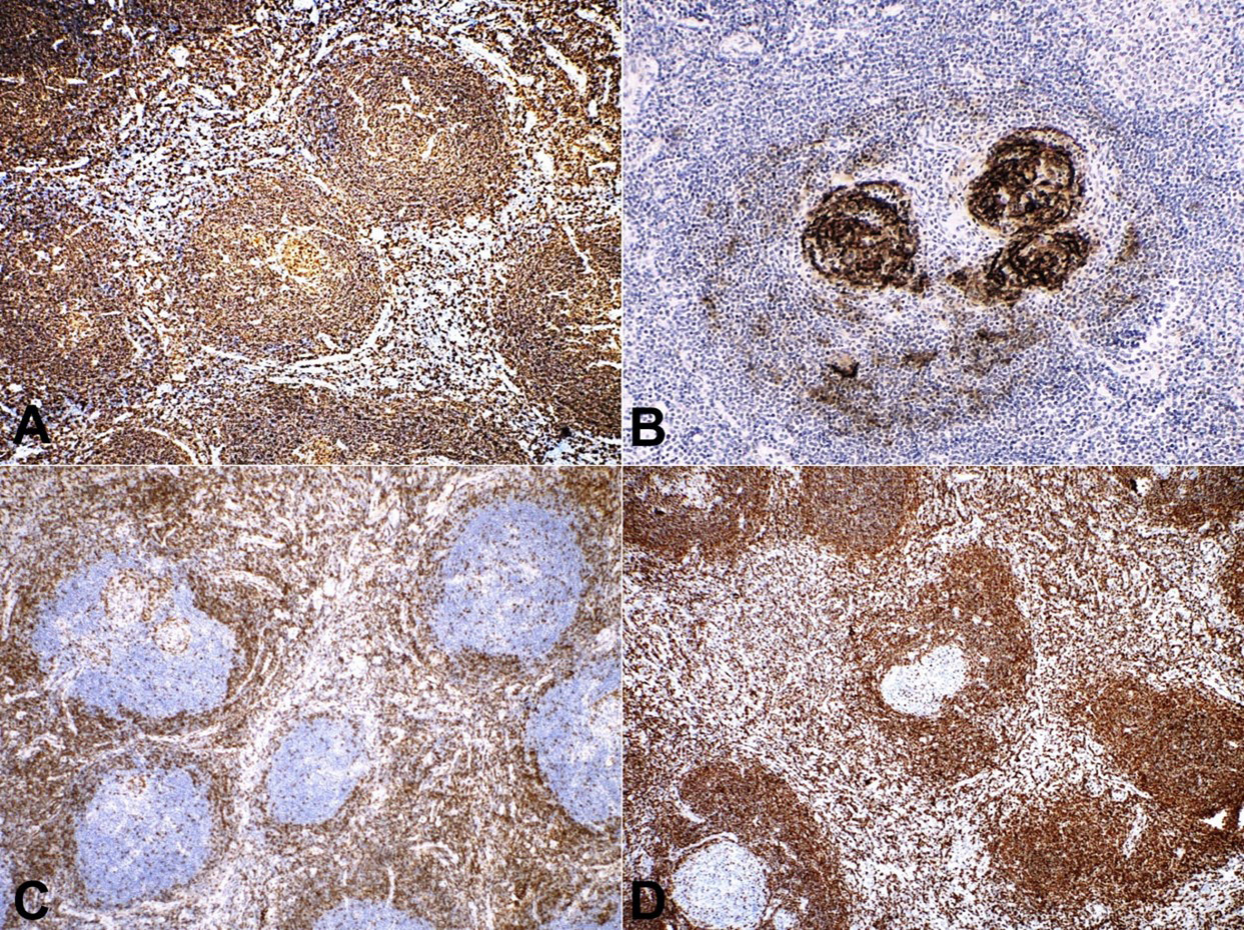
Immunohistochemical findings: **A** – Mature B cells showing positivity for CD20; **B** – FDCs showing positivity for CD21; **C** – CD3 positivity is seen in T cells; **D** – Bcl2 positivity is seen in non-germinal center B cells.

On immunohistochemistry, cells in the lymphoid follicles showed positive staining for CD20, a mature B cell marker (Figure 3A). FDCs in the abnormal germinal centers, interfollicular region, and within the concentric rims of lymphocytes were immunopositive for CD21 (Figure 3B). Interfollicular T cells were highlighted by CD3 (Figure 3C). Bcl-2 staining was positive in most B and T cells except those in the germinal centers (Figure 3D). Ki67 labeling index was high in the germinal centers.

## DISCUSSION

CD, also known as angiofollicular lymph node hyperplasia, was first described in the 1950s by Castleman and his colleagues in a group of patients with benign localized hyperplastic lymph nodes in the mediastinal area.^5,6^ It has an estimated prevalence of 16.2 per 100,000 population.^7^ The age of presentation ranges from 8 to 69 years, with no sex predilection.^8^ Our case was of a 63-year-old female presenting with left cervical lymphadenopathy. In the study by Mallik et al.^2^ and Ghosh et al.,^9^ all the cases had cervical lymph node involvement. CD can occur in a single lymph node (unicentric) or multiple lymph nodes (multicentric) associated with systemic symptoms. Patients with the unicentric disease generally present with a painless solitary mass usually localized to the mediastinum; however, the neck, axilla, abdomen, pelvis, and retroperitoneum are other common sites of occurrence.^10^ Multicentric CD involves multiple lymph nodes separately or in a confluent pattern. It is frequently associated with HHV-8 infection, Kaposi sarcoma, and HIV infection.^11^ The most common histologic subtype, hyaline-vascular CD, is characterized by germinal centers surrounded by concentric rings of mantle zone lymphocytes and the proliferation of hyalinized vessels in between the follicles. The plasma cell subtype of CD is characterized by paracortical plasmacytosis and plasmablasts in the mantle zone and paracortex. These patients usually present with multicentric disease and systemic symptoms such as fever, night sweats, and splenomegaly. Mixed cases have hyaline-vascular type follicles with increased plasma cells.^12^

As CD is a rare lymphoproliferative disorder, experience in the aspiration of this disease is limited to isolated case reports and small case series. In 1982, Hidvegi et al.^13^ were the first to describe its cytological features. To our knowledge, Ghosh et al.^9^ reported the largest series of 5 cases on the cytological features of hyaline vascular type of CD. The cytological diagnosis is challenging, and the smears are often misinterpreted as atypical or neoplastic. Ghosh et al.^9^ had previously diagnosed all of their 5 cases as reactive lymphadenitis. Both the FNA specimens in the series by Murro et al.^14^ were misdiagnosed as lymphoma. In their review of 3 cases, Mallik et al.^2^ reported one case as suspicious for Hodgkin lymphoma and the other two as atypical. Nevertheless, the awareness of the cytomorphology of the disease is essential for the appropriate management of the patients, as FNAC is generally the first modality of investigation undertaken in mass lesions. The cytological features of previously described cases of hyaline vascular type of CD are shown in Table 1.

**Table 1:** Cytomorphological features of previously reported cases of histologically confirmed hyaline vascular type of Castleman disease

Ref.	No. of cases	Age/sex	Clinical presentation	Key cytological features	Cytological diagnosis
Hidvegi et al.^13^	1	69/M	Retroperitoneal mass, 25cm	Cells: mainly small lymphocytes,	Consistent with CD
				FDCs: Occasional Capillaries: +	
				Others: Nil	
Sterrett et al.^15^	1	30/M	Anterior mediastinal mass	Cells: admixture of small lymphocytes and large lymphoid cells	D/D-Lymphoma, Thymoma
				FDCs: NS	
				Capillaries: -	
				Others: Nil	
Chan and McGuire^16^	1	43/F	Salivary gland swelling, 3.5 cm for 3 y	Cells: Mainly small lymphocytes	D/D – Benign lymphoid lesion, Warthin tumor
				FDCs: NS	
				Capillaries: +	
				Others: hyaline masses +	
Cangiarella et al.^17^	1	60/F	Mediastinal mass, H/o choking sensation for 3 mon	Cells: Monotonous population of small lymphocytes	Inconclusive
				FDCs: NS	
				Capillaries: -	
				Others: Clusters of cohesive large lymphoid cells	
Panayiotides et al.^18^	1	30/M	Left parotid lump, 4.5cm for 4 y	Cells: Small lymphocytes and few histiocytes	D/D - Benign lymphoepithelial lesion, Low-grade NHL
				FDCs: NS	
				Capillaries: -	
				Others: Many cohesive groups of salivary epithelial cells	
Meyer et al.^19^	2	1. 26/F	1. Right hilar mass, 3.5cm	Cells: Polymorphous population of lymphoid cells	Both cases: Atypical
				FDCs: +	
		2. 40/F	2. Parapharyngeal mass, 4.5cm, medial deviation of oropharynx	Capillaries: +	
				Others: Nil	
Taylor and Smeeton^20^	1	28/M	Paratracheal mass, 5cm, systemic symptoms	Cells: Polymorphous population, mainly small lymphocytes admixed with large lymphocytes, plasma cells, TBMs, and some atypical cells	Inconclusive
				FDCs: + (identified on RE)	
				Capillaries: -	
				Others: Nil	
Owens et al.^21^	1	34/M	POEMS syndrome, Left axillary LN, 2cm	Cells: Polymorphous mature lymphocytes	Hyperplastic lymph node
				FDCs: +	
				Capillaries: +	
				Others: Follicular	
				cell clusters with concentric arrangement of lymphocytes	
Mallik et al.^2^	3	1. 44/M	1. Mid-cervical mass, 2cm for 2 mon	Cells; Polymorphous population, lymphocyte predominance with interspersed large atypical cells	1. Suspicious for Hodgkin lymphoma
				FDCs: +	
			2. Supraclavicular mass, 3cm for 3 mon	Capillaries: +	2. Reactive with atypical cells
		2. 38/M		Others: Nil	
			3. Cervical mass, 4cm for 1 y		3. Atypical cytology
			
		3. 32/F			
Deschênes et al.^22^	1 (3 sites)	66/M	1^st^ – Left posterior cervical mass	Cells: Polymorphous lymphoid population	1^st^- Atypical
			2^nd^- FDG-avid lesion in right inguinal region	with occasional large histiocytes; predominance of large lymphocytes in 1^st^ FNA	2^nd^& 3^rd^- Benign/reactive with possible CD
			3^rd^- Enlarged left cervical lymph node	FDCs: Not seen	
				Capillaries: + in 2^nd^& 3^rd^ FNAs, and in 1^st^ on RE	
				Others: Nil	
Nanda et al.^23^	1	21/M	Left abdominal pain and mass, 7.2cm for 2 mon	Cells: Clusters of small lymphocytes	CD
				FDCs: +	
				Capillaries: +	
				Others: Nil	
Sudha and Vivekanand^24^	2	1. 38/F	1. Multiple left axillary LN	Cells: mixed population of small and large lymphoid cells and TBMs	Reactive hyperplasia with a strong indication of CD
		2. 25/M		FDCs: NS	
			2. Left cervical LN, 3cm for 3 mon	Capillaries: +	
				Others: Eosinophilic material within cell aggregates	
Ghosh et al.^9^	5	1. 32/F	1. 3cm left upper cervical mass for 6 mon	Cells: Polymorphous population, mainly small lymphocytes; one case showed few large cells	All 5 cases reported as reactive lymphadenitis
				FDCs: NS	
			2. 3 cm left mid cervical mass for 8 mon	Capillaries: + in all but one case	
		2. 26/F		Others: Nil	
			3. 5 cm right supraclavicular mass for 6 mon		
		
		3. 29/M	4. 2 cm right mid cervical mass for 1 y		
		
			5. 2 cm right mid cervical mass for 3 mon		
		4. 22/M			
		
		
		5. 19/F			
Khashab et al.^25^	1	27/F	4.2 × 4.3 cm, exophytic, hypervascular mass on CT	Cells: polymorphous lymphocytes with a predominance of B-lymphocytes on FCM	CD
				FDCs: NS	
				Capillaries: +	
				Others: variably sized and partially intact lymphoid follicles	
Gill et al.^26^	1	55/F	Painless, soft mass in the right arm for 2 y	Cells: polymorphous population, mainly small lymphocytes, few TBMs and few scattered large oval cells	Reactive hyperplasia with a strong indication of CD
				FDCs: NS	
				Capillaries: +	
				Others: Nil	
Malzone et al.^27^	1	44/M	5cm hard submandibular lump at scar site, 5 years after excision of submandibular lymph node	Cells: polymorphous population of lymphoid cells with numerous atypical lymphoid cells of medium to large-size	Atypical lymphoproliferative disease,
				FDCs: +	Possibility of CD considered
				Capillaries: +	
				Others: Atypical cells were CD30 negative and CD21 positive	
Murro et al.^14^	5	1. 47/M	1. Subcarinal LN	Cells: Mainly small lymphocytes	The two FNA cases were diagnosed as Hodgkin lymphoma and low-grade B-cell lymphoma respectively.
		2. 34/F	2. Right parotid	FDCs: Clusters +, with multinucleation, even chromatin, small nucleoli	
		3. 40/M	3. Left neck LN	Capillaries: +	
		4. 28/F	4. Mediastinal LN	Others: Large tissue fragments +	
		5. 63/F	5. Retro gastric LN		
		
Singh et al.^28^	1	2/F	5cm Right cervical mass for 1 y	Cells: polymorphous population of reactive lymphoid cells	Granulomatous lymphadenitis
				FDCs: NS (+ on RE)	
				Capillaries: NS (+ on RE)	
				Others: aggregates of plump spindle cells forming ill-defined granuloma-like structures (interpreted as endothelial cells on RE)	
Phulware et al.^29^	1	11/M	3.5x 3.5 cm right cervical lymphadenopathy	Cells: Mainly mature lymphocytes with large atypical mononuclear and binucleated cells showing large nuclei and distinct small nucleoli	Hodgkin lymphoma
				FDCs: NS	
				Capillaries: -	
				Others: Nil	
Chaurasia et al.^30^	1	19/M	1 cm preauricular swelling for 7 mon	Cells: Mixed population of lymphoid cells	NHL
				FDCs: NS	
				Capillaries: +	
				Others: Few eosinophils and plasma cells	
Singh et al.^31^	1	14/M	Multiple cervical lymphadenopathy	Cells: Mature and transformed lymphoid cells	Atypical lymphoid cells; suspicious of CD
				FDCs: + (Atypical)	
				Capillaries: +	
				Others: Few large atypical cells, occasional Warthin Finkeldey giant cells, eosinophilic material in germinal center fragments	
Bhatia et al.^32^	1	26/F	Pain abdomen and weight loss for 2 mon, FDG avid soft-tissue mass along the body of pancreas	Cells: Polymorphous population of lymphoid cells	HVCD
				FDCs: NS	
				Capillaries: +	
				Others: Eosinophilic material in cell aggregates	

M: Male; F: female; CD: Castleman disease; CT: Computed tomography; D/D: Differential diagnosis; FCM: Flowcytometry; FDC: Follicular dendritic cells; FDG: Fluor-deoxy-glucose; FNA: Fine needle aspiration; H/o: History of; HVCD: Hyaline vascular type of Castleman disease; LN: lymph node; Mon: Months; NHL: Non-Hodgkin lymphoma; NS: Not specified; POEMS: polyneuropathy, organomegaly, endocrinopathy, M-protein and skin changes; RE: Retrospective examination, Ref.: Reference; TBMs: Tingible body macrophages; Y: Years.

In our case, the cytosmears were cellular and comprised predominantly of small follicles along with a dispersed population of mature lymphocytes. The follicles had small germinal centers surrounded by concentric rims of small lymphocytes. The striking feature was the presence of scattered large follicular dendritic cells with mono-, bi- and multinucleated forms. These cells had finely granular chromatin with small nucleoli and abundant pale cytoplasm. Another distinct feature was the presence of capillary fragments within the follicles. Mallik et al.^2^ and Ghosh et al.^9^ have described highly cellular smears in all their cases. Large, atypical cells were described by Meyer et al.^19^ in all their cases, and by Ghosh et al.^9^ in only one of their 5 cases. Chan and McGuire^16^ did not find these large cells in their study. Mallik et al.^2^ reported the presence of large, atypical cells with “crumpled tissue paper” like chromatin, nuclear indentations, and nuclear grooves to be the most consistent clue for making the diagnosis of CD, a feature not seen in our case nor in the study by Ghosh et al.^9^ These large, atypical cells were immunopositive for CD21 and categorized as follicular dendritic cells.^20^ Mallik et al.^2^ have described capillary fragments in all their cases. In contrast, these were seen in 4 of the 5 cases in the study by Ghosh et al.^9^ Meyer et al.^19^ did not report capillary fragments in their study.

The differential diagnoses of hyaline-vascular CD include nonspecific follicular hyperplasia, Hodgkin lymphoma (HL), and Rosai-Dorfman disease. In reactive follicular hyperplasia, aspirates comprise a polymorphous population of predominantly small lymphocytes, scattered centroblasts, centrocytes, immunoblasts, plasma cells, tingible body macrophages, and dendritic lymphocytic aggregates.^14^ While CD also consists predominantly of small lymphocytes, the presence of large follicular dendritic cells and tissue fragments containing branching capillaries and/or hyaline material has been proposed as its cytologic hallmark. In HL, the background population is composed of mixed inflammatory cells, including eosinophils, lymphocytes, plasma cells, histiocytes, and neutrophils, while CD has a background population composed predominantly of mature lymphocytes. Large FDCs in CD can be confused with Reed-Sternberg (RS) seen in HL, but the two can be distinguished by macronucleoli and reticular chromatin seen in the latter.^20^ In Rosai-Dorfman disease, the key cytomorphologic feature is emperipolesis, i.e., the engulfment of lymphocytes by large histiocytes having pale nuclei and abundant vacuolated cytoplasm.^14^

## CONCLUSION

The present case highlights that FNA could be challenging, but a reliable cytological diagnosis of Hyaline vascular type of CD can be made based on three primary indicators: (i) the abundance of small lymphocytes present either individually or clustered together with follicular dendritic cells; (ii) the existence of a distinct population of enlarged follicular dendritic cells displaying varying degrees of atypia; and (iii) the observation of vessels traversing through lymphocytic clusters or seen as isolated fragments. The confirmation of follicular dendritic cells, which often show variable atypia, by their immunopositivity for CD21 and negativity for B and T cell markers, as well as for CD15 and CD30, is crucial to exclude the neoplastic etiology. Thus, cytology, in conjunction with immunocytochemical studies, can help the Pathologist arrive at the correct diagnosis and decide on the appropriate course of management.
